# P-1425. Burden of pneumococcal disease associated with PCV21 non-PCV20 and PCV20 non-PCV21 serotypes among US adults

**DOI:** 10.1093/ofid/ofaf695.1612

**Published:** 2026-01-11

**Authors:** Amanda M Martino, Laura DeBenedetti, Maria Vutcovici Nicolae, Sheba Nellore, Nicole Cossrow, Henok Tadesse Ayele, Kenneth Klinker, Kelly Johnson

**Affiliations:** Merck & Co., Inc., Rahway, NJ; Certara, Montreal, Quebec, Canada; Certara Canada, Montreal, Quebec, Canada; Certara, Montreal, Quebec, Canada; Merck & Co, Inc., Kenilworth, NewJersey; Merck & Co., Inc., Rahway, NJ; Merck & Co, Inc, Kenilworth, NJ; Merck & Co., Inc., Rahway, NJ

## Abstract

**Background:**

The introduction of pneumococcal conjugate vaccines (PCVs) into immunization schedules successfully reduced the burden of pneumococcal disease (PD) caused by vaccine serotypes in US adults. However, residual PD burden remains due to shifting serotype epidemiology from pediatric indirect protection and the emergence of non-vaccine serotypes. We aimed to quantify the residual PD burden in US adults addressable by PCV21 non-PCV20 and PCV20 non-PCV21 serotypes.

**Table 1.**
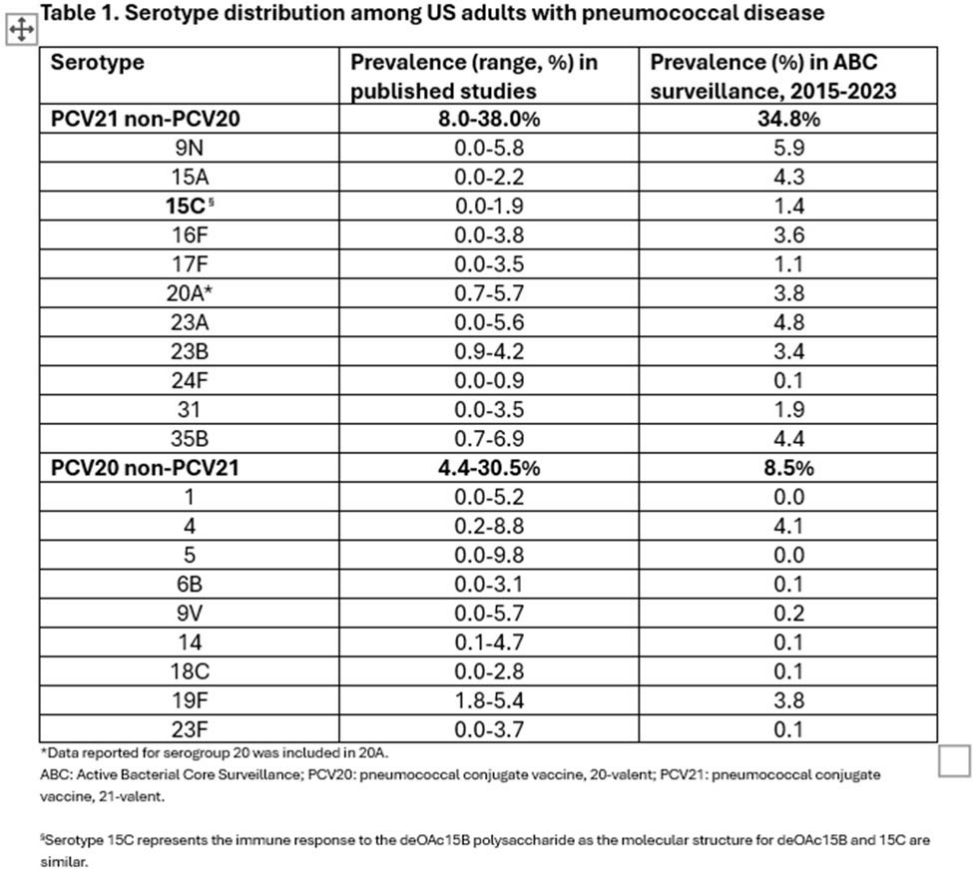
Serotype distribution among US adults with pneumococcal disease

**Methods:**

A systematic review of literature published from 2015 onwards was conducted to explore the epidemiology, antimicrobial resistance (AMR), and virulence associated with the PCV21 non-PCV20 serotypes (9N, 15A, 15C ^§^, 16F, 17F, 20A, 23A, 23B, 24F, 31, 35B); and the PCV20 non-PCV21 serotypes (1, 4, 5, 6B, 9V, 14, 18C, 19F, 23F) in the adult US population.

^§^Serotype 15C represents the immune response to the deOAc15B polysaccharide as the molecular structure for deOAc15B and 15C are similar.

**Results:**

In the 2015-2023 period, Active Bacterial Core Surveillance (ABC) data indicated that PCV20 non-PCV21 serotypes covered 8.5% of invasive pneumococcal disease (IPD) and PCV21 non-PCV20 serotypes covered 34.8%. Published studies providing full serotype distribution data (n=6) in adults reported a pooled prevalence of 8.0-38.0% and 4.4-30.5% for PCV21 non-PCV20 and PCV20 non-PCV21 serotypes, respectively (Table 1).

Among adults hospitalized for non-invasive PD (2009–2012), AMR was reported for seven unique serotypes in PCV21 non-PCV20 and one in PCV20 non-PCV21. PCV21 non-PCV20 serotypes had higher resistance to antibiotics commonly prescribed for non-invasive PD (penicillin: 96% vs 60%; erythromycin: 62% vs 50%) and lower resistance to antibiotics used to treat IPD (ceftriaxone: 9% vs 25%). In adults with invasive and non-invasive pneumonia (2009−2017), PCV21 non-PCV20 serotypes had higher resistance to erythromycin (89% vs 56% in PCV20) and lower resistance to penicillin (4% vs 33%) and ceftriaxone (5% vs 25%). Multidrug resistance rates were highest for serotypes 19F (42%) and 23A (27%).

**Conclusion:**

Among US adults with pneumococcal disease, PCV21 non-PCV20 serotypes are more prevalent than PCV20 non-PCV21 serotypes, and are associated with higher rates of resistance to antimicrobials used to treat non-invasive PD.

**Disclosures:**

Amanda M. Martino, MPH, Merck & Co., Inc.: Employee|Merck & Co., Inc.: Stocks/Bonds (Public Company) Laura DeBenedetti, n/a, Certara USA, Inc.: Employee of Certara, a consulting firm that received funding from Merck & Co., Inc. Maria Vutcovici Nicolae, MD MSc, Certara USA, Inc.: Employee of Certara, a consulting firm that received funding from Merck & Co., Inc. Sheba Nellore, MBA, Certara USA, Inc.: Employee of Certara, a consulting firm that received funding from Merck & Co., Inc. Nicole Cossrow, PhD, Merck & Co., Inc.: Employee|Merck & Co., Inc.: Stocks/Bonds (Public Company) Henok Tadesse Ayele, PhD, Merck & Co., Inc.: Employee|Merck & Co., Inc.: Stocks/Bonds (Public Company) Kenneth Klinker, PharmD, Merck: Employee Kelly Johnson, PhD, MPH, Merck & Co., Inc.: Employee|Merck & Co., Inc.: Stocks/Bonds (Public Company)

